# Jab1/COPS5 as a Novel Biomarker for Diagnosis, Prognosis, Therapy Prediction and Therapeutic Tools for Human Cancer

**DOI:** 10.3389/fphar.2018.00135

**Published:** 2018-02-27

**Authors:** Guohong Liu, Francois X. Claret, Fuling Zhou, Yunbao Pan

**Affiliations:** ^1^Department of Radiology, Zhongnan Hospital of Wuhan University, Wuhan University, Wuhan, China; ^2^Department of Systems Biology, The University of Texas, MD Anderson Cancer Center, Houston, TX, United States; ^3^Department of Hematology, Zhongnan Hospital of Wuhan University, Wuhan University, Wuhan, China; ^4^Department of Laboratory Medicine, Zhongnan Hospital of Wuhan University, Wuhan University, Wuhan, China; ^5^Breast Tumor Center, Sun Yat-Sen Memorial Hospital, Sun Yat-Sen University, Guangzhou, Guangdong, China

**Keywords:** Jab1, biomarker, prognostic marker, therapeutic target, COPS5

## Abstract

C-Jun activation domain-binding protein-1 (Jab1) involves in controlling cellular proliferation, cell cycle, apoptosis, affecting a series of pathways, as well as regulating genomic instability and DNA damage response (DDR). Jab1/COPS5 dysregulation contributes to oncogenesis by deactivating several tumor suppressors and activating oncogenes. Jab1 overexpression was found in many tumor types, illuminating its important role in cancer initiation, progression, and prognosis. Jab1/COPS5 has spurred a strong research interest in developing inhibitors of oncogenes/oncoproteins for cancer therapy. In this paper, we present evidences demonstrating the importance of Jab1/COPS5 overexpression in several cancer types and recent advances in dissecting the Jab1/COPS5 upstream and downstream signaling pathways. By conducting ingenuity pathway analysis (IPA) based on the Ingenuity Knowledge Base, we investigated signaling network that interacts with Jab1/COPS5. The data confirmed the important role of Jab1/COPS5 in tumorigenesis, demonstrating the potential of Jab1/COPS5 to be used as a biomarker for cancer patients, and further support that Jab1/COPS5 may serve as a potential therapeutic target in different cancers.

## Introduction

Jab1/COPS5 was initially identified as c-Jun activation domain-binding protein-1 (Jab1) (Claret et al., 1996), a c-Jun coactivator, and subsequently discovered to be the fifth component of the constitutive photomorphogenic-9 signalosome (COPS5, CSN5), a multifunctional protein implicated in regulating a wide variety of cellular and developmental processes, including signal transduction, cells proliferation, cell cycle, apoptosis, DNA damage response (DDR), and tumorigenesis (Lee M. H. et al., 2011). Jab1/COPS5 gene is localized at chromosome 8Q13.2 and encodes Jab1 protein with a molecular mass of 38 kDa. Jab1/COPS5 contains a c-Jun binding domain (JBD), an Mpr1-Pad1-N-terminal (MPN) domain containing the Zn^2+^-metalloprotease motif (JAMM) that provides the catalytic center to the complex for the CSN isopeptidase activity (Ambroggio et al., 2003; Wei and Deng, 2003; Lingaraju et al., 2014), a nuclear export signal (NES) domain, and a p27 binding domain (PBD) at the C-terminal end. Increasing evidence indicates that dysregulation of Jab1/COPS5, which functionally interacts with several tumor-related genes, such as the cyclin-dependent kinase (CDK) inhibitor 1B p27 (Tomoda et al., 1999; Sui et al., 2001; Esteva et al., 2003), transformation-related phosphoprotein p53 (Zhang et al., 2008), cell signaling protein mothers against decapentaplegic homolog 4/7 (SMAD4/7) (Li et al., 2015), transcriptional coregulatory protein nuclear receptor co-repressor (NcoR) (Lu et al., 2016), Cyclin-dependent kinase inhibitor 1C (p57) (Guo et al., 2016), transmembrane protein Programmed death-ligand 1 (PD-L1) (Lim et al., 2016), and so on, contributes to tumorigenesis. Jab1/COPS5 as an oncogene, is aberrantly overexpressed in various human cancers. However, the mechanism by which Jab1/COPS5 facilitates cancer progression remains largely elusive. Herein, we review advances in understanding the oncogenic role of Jab1/COPS5 in tumorigenesis as well as its therapeutic implications in combating cancer.

## Basic function of Jab1/COPS5

### Structure-based role of Jab1/COPS5

#### Binding protein

Jab1/COPS5 is a key COP9 signalosome (CSN) subunit, which is able to integrate multiple functions of the CSN complex. The CSN was originally characterized as a retardant of plant photomorphogenesis (Wei and Deng, 1992). It contains eight subunits, CSN1 to CSN8 (Gusmaroli et al., 2007). Among them, 6 subunits contain a PCI (proteasome-COP9 signalosome-initiation factor 3) domain that serves as a structure scaffold of the assembly of CSN, while only 2 subunits contain an MPN (MPR1-PAD1-N-terminal) domain (Wei et al., 2008). Jab1/COPS5 contains a nuclear export signal (NES) domain and a p27 binding domain (PBD) at the end of the C-terminal (Shackleford and Claret, 2010). Through the binding of P27 to PBD, Jab1/COPS5 carries p27 protein out of cell nucleus in a Exportin 1 (XPO1)-dependent manner through the NES sequence between amino acids 233 and 242 at its C-terminal end (Shackleford and Claret, 2010; Wang L. et al., 2016).

#### Isopeptidase activity

While both Csn5 and Csn6 contain an MPN domain, only Csn5 has an embedded JAMM (JAB1 MPN domain metalloenzyme) motif, which takes effect as the catalytic center for CSN isopeptidase activity (Tran et al., 2003; Wei et al., 2008; Echalier et al., 2013). The MPN domain of Jab1/COPS5 is responsible for regulating the cullin deneddylation process by activating the CRL (Cullin-RING family of ubiquitin ligases) activity (Schwechheimer and Deng, 2001; Wolf et al., 2003; Duda et al., 2008). The MPN domain stands for a pseudo enzyme domain that has been converted into a protein-protein interaction platform which has been confirmed by experimental results (Rosel and Kimmel, 2006; Busch et al., 2007). Adler et al. showed evidence that the isopeptidase activity of Jab1/COPS5 was critical for human and mouse mammary epithelial transformation and progression (Adler et al., 2008).

### Jab1/COPS5 inside or independently outside the CSN

Jab1/COPS5 is a pivotal CSN subunit, which is able to integrate various functions of the CSN complex (Chamovitz and Segal, 2001). As a unique subunit of the CSN, COPS5 harbors the catalytic center of the CSN isopeptidase activity and also steadily exists independent of CSN complex *in vivo* (Wei and Deng, 2003). Jab1/COPS5 energetically takes part in crucial biological activities, both as a part of the CSN complex and independent of the CSN complex. A large portion of Jab1/COPS5 is discovered in free form, which seem to be located in both cytoplasm and nucleus (Wei and Deng, 2003; Wei et al., 2008), while the CSN-associated Jab1/COPS5 is mainly in nuclear. The multi-functionality of Jab1/COPS5 originates from the fact that it exists as a morphon or a sub-complex out of the integral CSN complex, and it was suggested as an individual factor or the central active component of the CSN complex (Kwok et al., 1998; Oron et al., 2002; Sharon et al., 2009). The CSN modulation of the CRL family of ubiquitin E3 complexes depends on its deneddylation function, which is fulfilled by removing NEDD8/Rub1 (an ubiquitin-like molecule) from the cullin subunit of cullin-containing E3 ligases (Cope and Deshaies, 2003; Dubiel, 2009). The JAMM of Jab1/COPS5 seems critical to the deneddylation activity of CSN and is essential for Jab1/COPS5's coactivation of MYC as well as Jab1/COPS5's transformative effects in breast epithelial model which is also proved to be dependent on the whole CSN (Sharon et al., 2009). The CSN is a multi-subunit complex that regulates protein stability by modulating the CRL family and acts as a regulator in several cellular processes, such as gene transcription, cell cycle, and DDR (Cope and Deshaies, 2003; Adler et al., 2006; Wei et al., 2008; Chamovitz, 2009; Kato and Yoneda-Kato, 2009). Other than being a CSN's catalytic center that is required for the deneddylase activity, Jab1/COPS5 alone has no metalloproteinase activity, and other CSN components, or maybe the whole complex, are needed for this deneddylase activity (Cope et al., 2002; Cope and Deshaies, 2003). The nuclear accumulation of Jab1/COPS5 dependent on other CSN components has been clearly demonstrated in the CSN-like complex of budding yeast (Maytal-Kivity et al., 2002). Although Jab1/COPS5 locates in both nuclear and cytoplasmic, whether Jab1/COPS5 acts independently or as part of the CSN complex in cancers needs further investigation (Pan and Claret, 2012).

## Jab1/COPS5 overexpression in human cancer

### Jab1/COPS5 overexpressed in cancer

**Figure 1:**
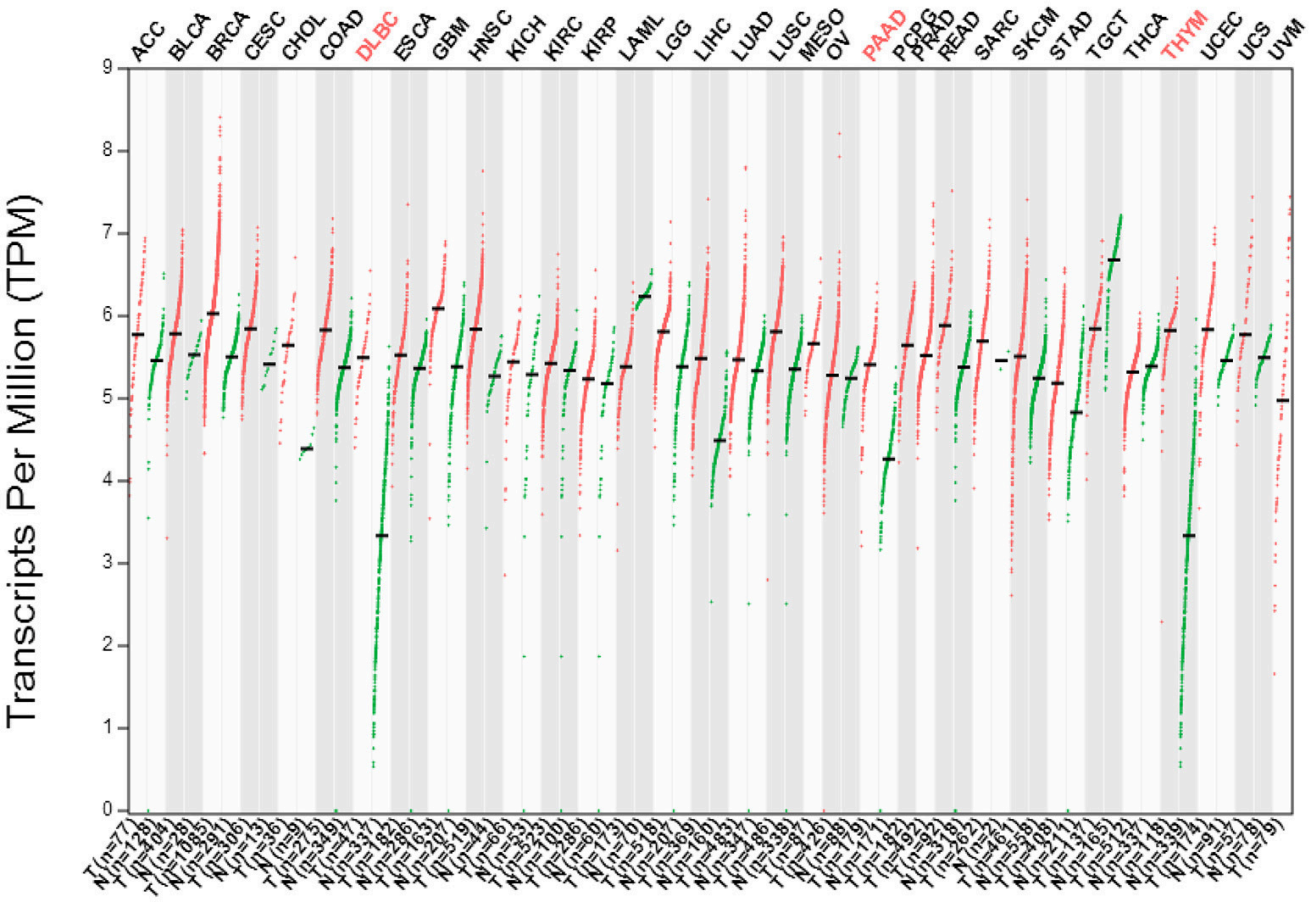
The gene expression profile across all tumor samples (T) and paired normal tissues (N) (Dot plot). Each dot represent expression level of samples. Data was downloaded from GEPIA database (http://gepia.cancer-pku.cn/index.html).

Cancer is a progressive disease which usually results from genomic instability that could be caused by chromosomal translocations, which leads to aberrant expression of oncogenes, such as *Jab1/COPS5* or *MYC*. Here we searched the GEPIA database (http://gepia.cancer-pku.cn/index.html) to systematically assess the differential expression of Jab1 in a variety of carcinomas. As demonstrated in Figure 1, while Jab1/COPS5 is systemically expressed in both tumor tissues and normal tissues, Jab1/COPS5 expression levels in cancers are significantly higher than those in normal tissues. Actually, Jab1/COPS5 overexpression was reported in many kinds of cancers, including hepatocellular carcinoma (HCC) (Hsu et al., 2008b), pancreatic cancer (Kouvaraki et al., 2006), breast cancer (Kouvaraki et al., 2003), non-small cell lung cancer (NSCLC) (Osoegawa et al., 2006), nasopharyngeal carcinoma (NPC) (Pan et al., 2012), and many others (Pan and Claret, 2012; Pan et al., 2014).

Breast cancer patients without Jab1/COPS5 expression did not recur or progression (Kouvaraki et al., 2003). Besides, Jab1/COPS5 was overexpressed in ovarian tumors and correlated with poor overall survival (Sui et al., 2001). Interestingly, overexpression of Jab1/COPS5 was positively associated with HCV infection and negatively correlated with HBV infection in HCC, indicating a possible mechanism that promotes hepatocarcinogenesis (Hsu et al., 2008b). However, there was no statistically significant relationship between Jab1/COPS5 and clinicopathological parameters or patient survival in intrahepatic cholangiocarcinomas and HCC (Berg et al., 2007; Hashimoto et al., 2009). Among NSCLC, patients with high Jab1/COPS5 expression levels have poor outcomes with a 5-year overall survival rate of 43.9%, compared with 63.1% for patients who have low level of Jab1 expression (Osoegawa et al., 2006). Researchers also showed that Jab1/COPS5 expression was closely linked to histological differentiation, lymph node metastasis, and clinical stage in oral squamous cell carcinoma (Gao et al., 2012). Patients with Jab1/COPS5 overexpression tended to have larger tumor size in thyroid carcinoma and poor overall survival in oral squamous cell carcinoma, NPC, and laryngeal squamous cell carcinomas, indicating its critical role in head and neck cancer (Dong et al., 2005; Ahn et al., 2009; Gao et al., 2012; Xu et al., 2015). Furthermore, Jab1/COPS5 was involved in chemotherapy and radiotherapy resistance (Pan et al., 2013b). Suppression of Jab1/COPS5 made NPC cells more sensitive to cisplatin and ionizing radiation (Pan et al., 2013b). Impressive progress has been made in deciphering the critical role of Jab1/COPS5 in diverse cellular and developmental processes.

### Why Jab1/COPS5 overexpressed in cancer?

Three regulatory mechanisms may contribute to the dysregulated expression of Jab1/COPS5 in cancer.

**Figure 2:**
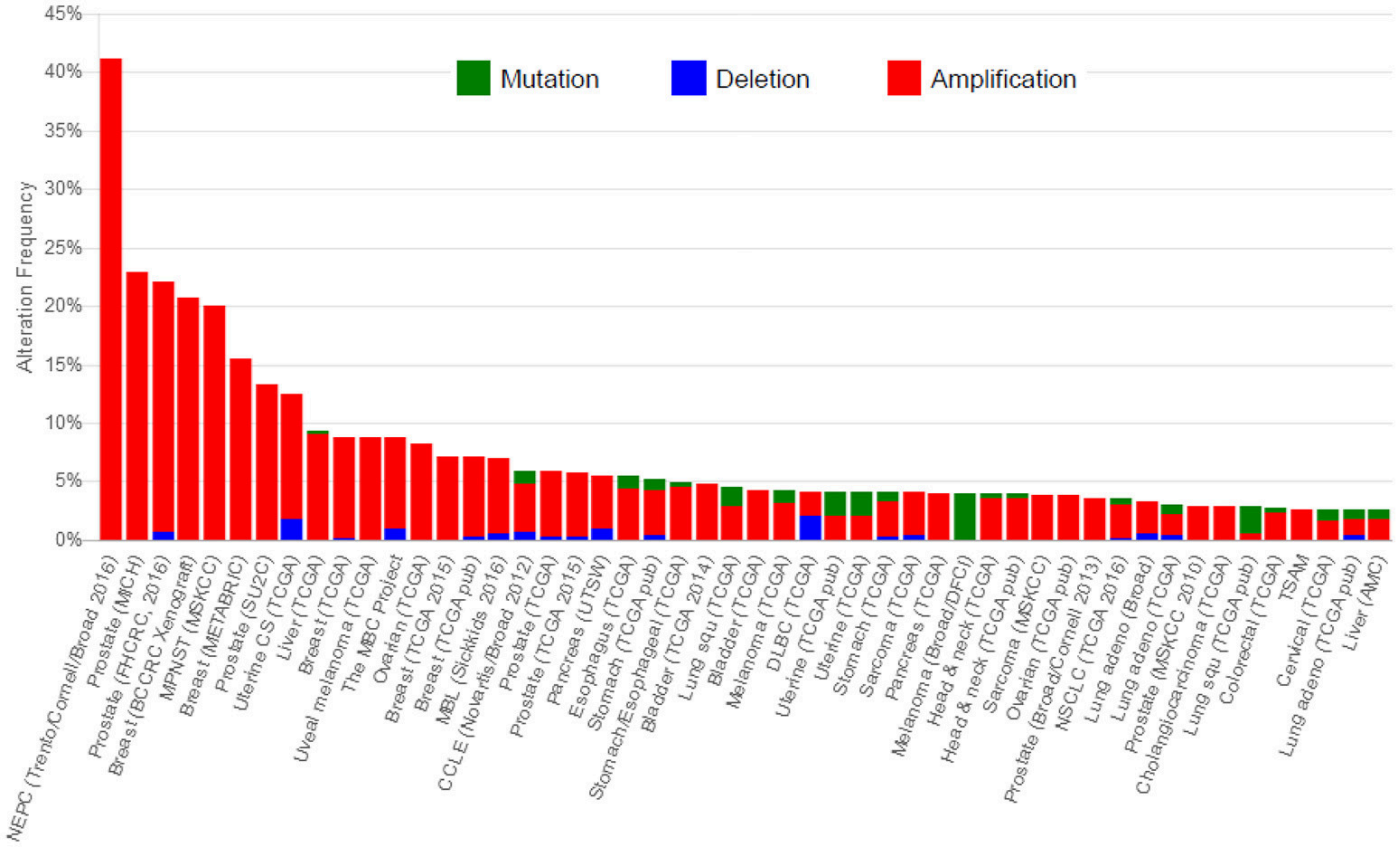
Cross-cancer alteration summary for COPS5. Data was downloaded from cBioportal (http://www.cbioportal.org/).

First, overexpression of Jab1/COPS5 may originate from gene amplification. The *Jab1/COPS5* locus which locates on chromosome 8q13.1, is found to be frequently amplified in human cancers (Dimova et al., 2009; Lu et al., 2016). We did a statistics of DNA alteration frequency of Jab1 in various cancer types on cBioportal database, which is shown in Figure 2. Data downloaded from cBioportal shows that 41.4, 22.9, and 20.7% genetic alterations of Jab1/COPS5 have been recorded in neuroendocrine prostate cancer (NEPC), colorectal adenocarcinoma triplets (MSKCC) and breast cancer, respectively.

Second, miRNAs may regulate Jab1 expression. Our group recently, discovered that miR-24 interacted with both the 3′UTR and 5′UTR of Jab1, resulting in Jab1 mRNA degradation and translational suppression. Although miRNAs always target the 3′UTR of the target gene (Lal et al., 2009), miR-24 could target both the 3′UTR and 5′UTR of Jab1 (Wang S. et al., 2016). Furthermore, miR-24 levels inversely associated with Jab1 mRNA and protein levels in both NPC cells and patients (Wang S. et al., 2016). Additionally, Jab1/COPS5 expressed at a higher level in recurrent NPC tissue than the matched primary tissue from the same patients. Jab1/COPS5 overexpression is correlated with a short duration from initial treatment to NPC recurrence (Wang S. et al., 2016). miR-24-Jab1/COPS5 axis represents a novel biomarker for NPC.

Third, other signaling pathways may contribute to Jab1/COPS5 overexpression, such as IL6-Stat3 signaling, HER2-AKT signaling, and Bcr-Abl signaling, which is described below. Moreover, Psoriasin (S100A7), a small calcium-binding protein, enhances Jab1/COPS5 activity as well as AP-1 activity, and promotes tumorigenesis (Emberley et al., 2003).

The mechanism of Jab1/COPS5 dysregulation in cancer patients still needs further exploration, but at least we can conceive that Jab1/COPS5 is a promising biomarker for cancer.

## Jab1/COPS5 associated signaling pathways and targets in cancer

**Figure 3:**
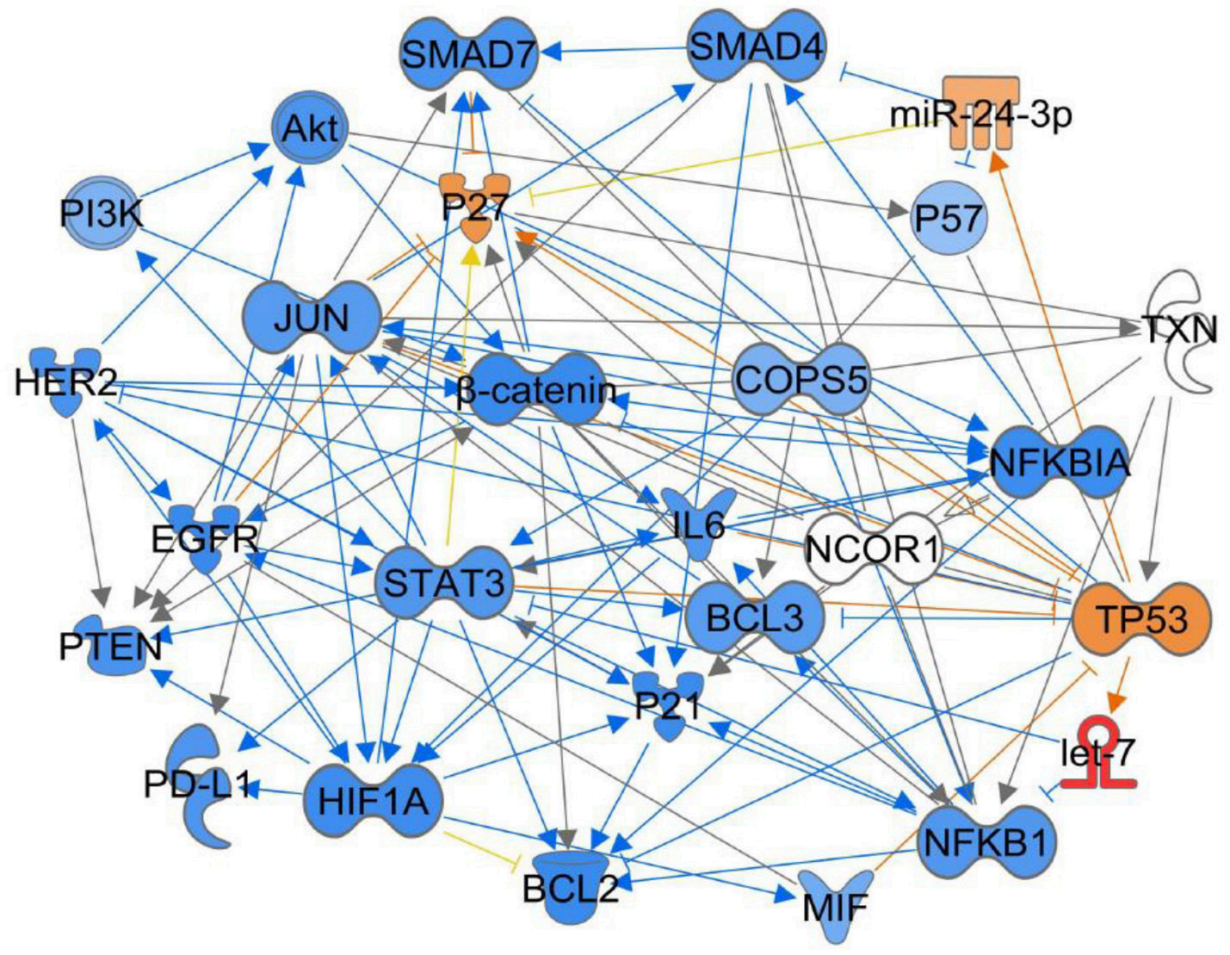
Ingenuity Pathway Analysis (IPA) for Jab1/COPS5 regulation network in cancer. Jab1/COPS5 is a multiple protein and plays an essential role in tumorigenesis. In this case we showed the predicted expression level of other genes in the regulation network in response to increased measured level of let-7 based on the IPA database. The color of each gene represents the corresponding expression level. Light-red means activation and light-blue means inhibition, while red means increased measurement and green means decreased measurement.

Jab1/COPS5 lies at the intersection of many signaling pathways that are believed to play important roles in tumorgenesis. Finding out the role of Jab1/COPS5 in these signaling pathways may help us to understand tumorigenesis. Specifically, we performed ingenuity pathway analysis (IPA) based on the Ingenuity Knowledge Base to understand the Jab1/COPS5 related network (Figure 3), we will also discuss Jab1/COPS5 related targets in the following section.

### Upstream signaling pathways

#### MiR-24-Jab1 signaling

**Figure 4:**
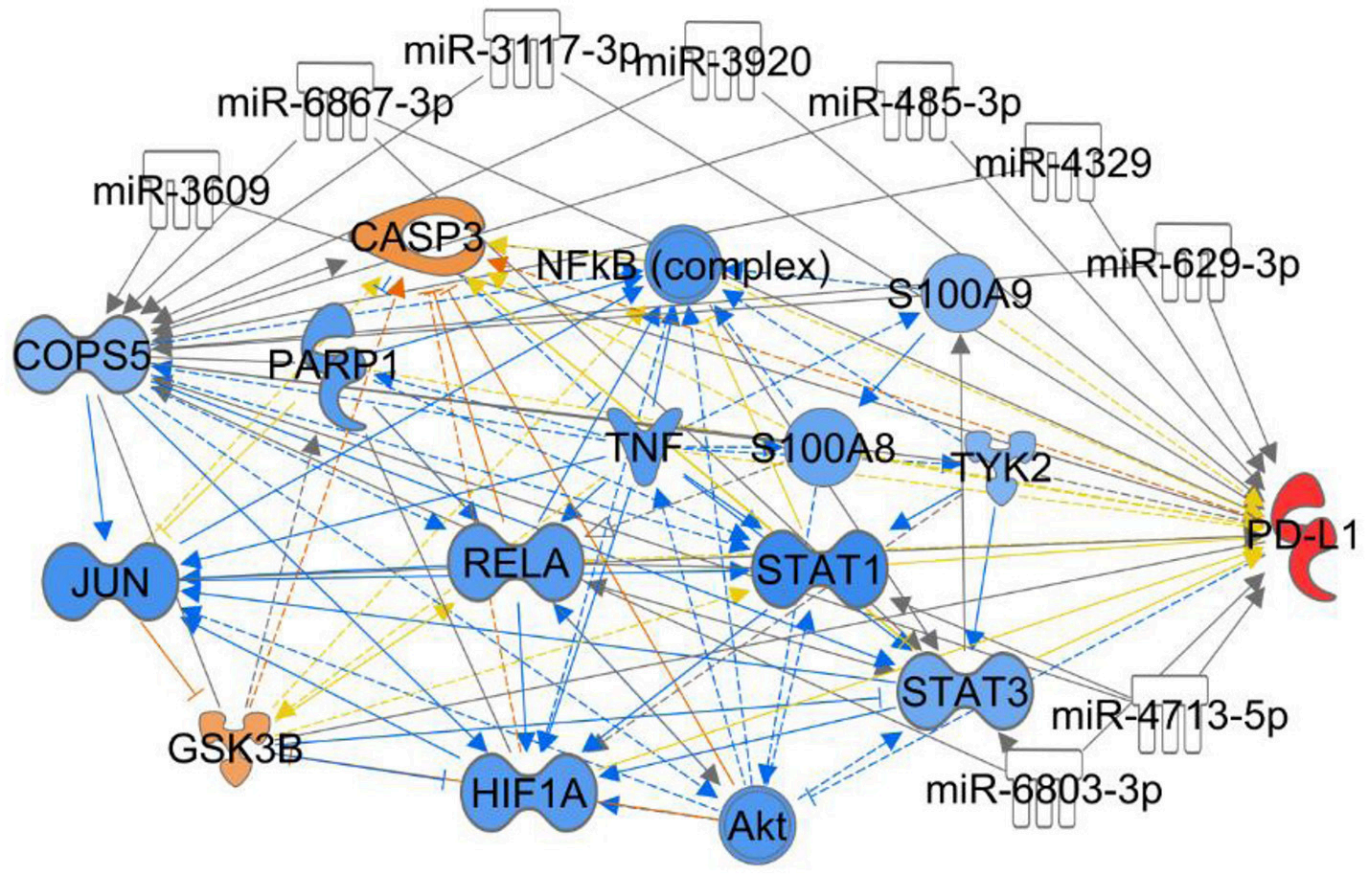
Ingenuity Pathway Analysis for the interaction between Jab1/COPS5 and PD-L1. In this case we showed the predicted expression level of other genes in the regulation network in response to increased measured level of PD-L1 based on the IPA database. The miRNAs in the figure are predicted ones that can target both Jab1/COPS5 and PD-L1, but no demonstration was reported yet. The color of each gene represents the corresponding expression level. Light-red means activation and light-blue means inhibition, while red means increased measurement and green means decreased measurement.

miR-24 functions as tumor suppressor and radiosensitizer in NPC cells and xenografts by inhibiting Jab1 translation through targeting both the 3′ untranslated region (3′UTR) and 5′UTR of Jab1, leading to tumor growth inhibition, and sensitizes NPC tumors to radiation *in vivo* (Wang S. et al., 2016). For other predicting miRNAs shown in Figure 4, their associations with cancer are waiting to be investigated.

#### IL6-stat3 signaling

Jab1/COPS5 regulates DNA-binding activity of unphosphorylated Stat3 through protein–protein interaction. Knockdown of Jab1/COPS5 significantly induced reduction of unphosphorylated Stat3 DNA-binding activity and Stat3 target genes, but nuclear Stat3 level was found increased in human colon cancer cell (Nishimoto et al., 2013). This non-intuitive phenomenon needs further investigation to discover how Jab1/COPS5 determines the fate of the transcription factors.

Our studies also revealed that Stat3 functions by binding to Jab1/COPS5 promoter, promotes its activity and increases Jab1/COPS5 transcription. Knockdown of Stat3 greatly impairs Jab1/COPS5 promoter activity and decreases Jab1/COPS5 RNA and protein levels (Shackleford et al., 2011; Pan et al., 2017b). Besides, upstream activators of Stat3, such as IL-6 and Src, also promote the activation of Jab1/COPS5 transcription and translation through interacting with Stat3.

#### HER-2 and EGFR signaling

It is reported that overexpression of Jab1/COPS5 is associated with human epidermal growth factor receptor-2 (HER-2) levels in breast cancer (Hsu et al., 2008a). HER-2 enhances Jab1/COPS5 promoter activity and increases Jab1/COPS5 expression through AKT/β-catenin pathway (Hsu et al., 2007). Through Inhibition of HER-2 by herceptin, Jab1/COPS5 expression was attenuated in various breast cancer cell lines (Le et al., 2005). In addition, Jab1/COPS5 is also a target in epidermal growth factor receptor (EGFR) pathway. Jab1/COPS5 expression level associates with EGFR level in breast cancer. Activation of EGFR promotes the translocation of Jab1/COPS5 from the cytoplasm to the nucleus and regulates p27 which is a representative gene downstream of Jab1/COPS5 (Wang et al., 2008).

#### TGF-β signaling

Jab1/COPS5 affects TGF-β signaling activity by specifically controlling the subcellular localization and degrading two key down-stream targets, Smad4 and Smad7 (Wan et al., 2002; Kim et al., 2004). By binding to Smad4, the receptor-regulated Smad protein (R-Smads) translocates into the nucleus and activates transcription of various target genes. Smad7 inhibits responses mediated by TGF-β and acts in a negative feedback loop to regulate the intensity or duration of the TGF-β signal. Jab1/COPS5 directly binds to Smad4 and degrades it through the proteasome pathway and thus attenuate TGF-β mediated gene transcription, whereas its degradation of Smad7 results in enhanced TGF-β signaling effects.

#### Wnt signaling

Jab1/COPS5 controls the Wnt/β-catenin signaling by assisting the formation of a β-catenin-degrading super-complex through deneddylation and simultaneous stabilization of adenomatous polyposis coli (APC) by CSN-associated deubiquitinylase, ubiquitin specific peptidase 15 (USP15) (Huang et al., 2009). Disturbance of the balance between β-catenin and APC can cause cancer by driving cell transformation, tumor angiogenesis and metastasis. Additionally, a clear-cut reduction in β-catenin levels as well as its phosphorylated form has been observed in Jab1/COPS5 knock-down colorectal cancer cells, indicating that Jab1/COPS5 plays an important role in regulating β-catenin and the associated Wnt pathway (Schutz et al., 2012).

#### NF-κB signaling

Jab1/COPS5 has also emerged as a regulator of NF-κB signaling by interacting with the proto-oncogene Bcl-3, a member of the Iκ B family that is present predominantly in the nucleus. While most members of the Iκ B family (IκBα, IκBβ, and IκBκ) act to inhibit NF-κB, Bcl-3 on the other hand, can activate NF-κB transcription. Jab1/COPS5 enhances NF-κB p50-Bcl-3-DNA complex formation and coactivates Bcl-3 stimulated NF-κB transcription through interacting with Bcl-3 (Dechend et al., 1999). A distinct cross-talk between NF-κB pathway and CSN-signalosome was then confirmed by the fact that Jab1/COPS5 controls NF-kB activity through CSN-associated deubiquitinase USP15, which causes deubiquitination of IκBα, a negative feedback between degradation of IκBα and activation of NF-κB (Schweitzer et al., 2007). Additionally, Jab1/COPS5 may be regulated by CSN-interacting kinases such as IκB kinase (IKK) (Orel et al., 2010).

#### MIF-PI3K-AKT signaling

An indirect mechanism between Jab1/COPS5 and PI3K/AKT pathway has been identified that Jab1/COPS5 inhibits MIF (autocrine macrophage migration inhibitory factor) secretion and its autocrine pro-survival activities and subsequently controls MIF mediated activation of PI3K/AKT signaling (Lue et al., 2007). In contrast, MIF also negatively regulates Jab1/COPS5 by specifically interacting with Jab1/COPS5 and inhibiting the activity of activator protein 1 (AP-1) and c-Jun amino terminal kinase (JNK) (Kleemann et al., 2000).

#### Bcr-Abl signaling

Bcr-Abl regulates Jab1/COPS5 by the cooperative interaction with β-catenin and Stat1 in leukemia cells. Suppression of Bcr-Abl by imatinib decreases Jab1/COPS5 expression in Bcr-Abl-positive leukemia cells (Yang et al., 2011). Besides, PI3K/Akt may participate in the regulation of Jab1/COPS5 by Bcr-Abl since PI3K/Akt inhibitor LY294002 decreases the promoter activity and mRNA level of Jab1/COPS5 (Tomoda et al., 2005).

### Jab1/COPS5 related targets

#### Jab1/COPS5-p27 axis

A growing body of evidence has interpreted that overexpression of Jab1/COPS5 is negatively correlated with p27 level and associated with worse prognosis in a variety of human cancers (Shackleford and Claret, 2010). P27 is deemed to be a primary modulator of cell cycle progression (Shackleford and Claret, 2010), and leads to cell cycle arrest in G1 phase and eventually suppress cell proliferation. Jab1/COPS5 directly combines with p27 and shuttles nuclear p27 to cytoplasm through NES (Zhang et al., 2008). Furthermore, p27's cytoplasmic translocalization has been noticed in human cancers and is tightly correlated with poor survival (Guo et al., 1997; Loda et al., 1997; Masciullo et al., 1999). Many studies demonstrated that Jab1/COPS5 increased the p27 nuclear translocation, and subsequent accelerated p27 degradation through the ubiquitin-proteasome pathway (Pan et al., 2012).

#### Jab1/COPS5- PD-L1 axis

The association between Jab1/COPS5 and PD-L1 was first reported by Mien-Chie Huang's group. They found that inflammation increases PD-L1 expression in tumors through TNF-α-mediated activation of NF-kB, resulting in transactivation of Jab1/COPS5. Jab1/COPS5 reduces PD-L1 ubiquitination and stabilizes it. Jab1/COPS5 enzyme activity controls T cell suppression via PD-L1. Further, inhibition of Jab1/COPS5 cooperates with anti-CTLA4 to enhance anti-tumor T cell function and decrease tumor growth (Lim et al., 2016). Although, IPA indicates multiple targets involves in Jab1/COPS5 and PD-L1 network (Figure 4), further studies need to be performed to elicit the regulatory mechanism.

#### Jab1/COPS5- NcoR axis

Jab1/COPS5 involves in breast cancer progression by regulating a variety of targets. NCoR is a molecular target of Jab1/COPS5 which mediates endocrine-resistance in breast cancer (Lu et al., 2016). Lin Guo et al. observed a remarkable reduction of NcoR protein expression in the tamoxifen-resistant MCF7 clones while Jab1/COPS5 is overexpressed, indicating a functional interaction between Jab1/COPS5 and NcoR complex. It was also confirmed that Jab1/COPS5 binds to NcoR. Intriguingly, while Jab1/COPS5 is significantly over-expressed in refractory tumors, NCoR is under-expressed in tamoxifen-resistant breast tumors. It is also confirmed that overexpression of Jab1/COPS5 degrades NCoR protein through the ubiquitination-proteasome pathway in breast cancer (Lu et al., 2016).

#### Jab1/COPS5-p57 axis

P57 is a member of CKIs family that also includes p21 and p27 (Matsuoka et al., 1995). P57 plays a role in cell proliferation, apoptosis, differentiation, and cell migration (Zhang et al., 1997; Matsumoto et al., 2014). Depletion of Jab1 stimulated Smad 2/3 phosphorylation and decreased CDK6 and cyclin D1 (Lee Y. H. et al., 2011). It has also been reported that cyclin D1, Smad, and TGF-β have cross-talk with p57 in cell functions (Zou et al., 2011). Our group recently evaluated Jab1 and p57 levels in HCC and found that Jab1 is inversely correlated with p57 in HCC tissues (Guo et al., 2016). Further studies revealed that Jab1 promotes p57's degradation through 26S proteasome pathway (Guo et al., 2016). We also observed that Jab1 physically interacts with p57 and p57 proteolysis occurred independently of Skp2 and Akt pathways in HCC cells (Guo et al., 2016). Importantly, downregulation of Jab1 sensitized HCC cells to cisplatin (Guo et al., 2016), indicating that Jab1/p57 pathway render chemotherapy resistance and may represent a potential target for treatment of HCC.

#### Jab1/COPS5-MDM2-p53 axis

Jab1/COPS5 is a critical modifier for both MDM2 and p53 (Zhang et al., 2008). It was demonstrated that p53's function is lost in almost half of human cancers (Nigro et al., 1989) and that maintaining the stability of p53 is fateful for its tumor suppressor function. Murine double minute 2 (MDM2), a ring domain-containing E3 ubiquitin ligase for anti-oncogene p53, can debase the half-life and stability of p53 through the ubiquitin-proteasome pathway (Iwakuma and Lozano, 2003). It is found that Jab1/COPS5 has a dual effect in decreasing the stability of p53 and facilitating MDM2-mediated p53 degradation. Jab1/COPS5 can also hinder MDM2 mediated self-ubiquitination (Fang et al., 2000), as a consequence, stabilizing MDM2 protein, which in reverse promoted p53 ubiquitination (Zhang et al., 2008). Jab1/COPS5 can implement its biological function by insulating its target proteins, and the most iconic and persuasive example is that Jab1/COPS5 triggers p27 protein translocating from cell nucleus to cytoplasm and subsequently prompts p27 degradation (Tomoda et al., 1999). Jab1/COPS5 is efficient in accelerating p53 nuclear export (Zhang et al., 2008). However, it is not explicit how Jab1/COPS5 can stabilize MDM2.

The above studies have revealed the crosstalk between Jab1/COPS5 and well-known signaling pathways and oncogenes and suggested that Jab1/COPS5 participated in cancer progression. Figure 3 generalizes some of Jab1/COPS5 related signaling pathways in human cancer.

#### Jab1/COPS5- Bcl2 axis

The Bcl-2 family of proteins is the key regulator of cell apoptosis at the mitochondria level and predominantly acts as an upstream of irreversible cellular damage in the intrinsic apoptotic pathway (Adams and Cory, 1998; Reed, 1998; Gross et al., 1999). Bcl-2 family contains both proapoptotic and anti-apoptotic members, all of which contain at least one of four conserved Bcl-2 homology (BH) domains, designated BH1–BH4 (Adams and Cory, 1998; Gross et al., 1999). BclGs (Bcl-Gonad short form), originally identified as an alternative mRNA splicing short form of BCL-G gene (Guo et al., 2001), is specifically highly expressed in human testis. Apoptosis induced by BclGs is dependent of the BH3 domain and suppressed by co-expression of anti-apoptotic Bcl-XL/Bcl-2 protein (Guo et al., 2001). Bcl-XL/Bcl-2 was also co-immunoprecipitated with BclGs but not with mutants of BclGs (Guo et al., 2001). Liu X, et al. have screened proteins that interact with BclGs in a human testis cDNA library (Liu et al., 2008) and found that Jab1 can regulate mitochondria apoptosis pathway via its interaction with BclGs. Jab1 binds to the N-terminal region of BclGs (aa 1–67) but not the BH3 domain and competes with Bcl-XL/Bcl-2 to bind to BclGs; thus, promotes apoptosis (Liu et al., 2008).

Bax is a pro-apoptotic member of the Bcl-2 family proteins which serve as critical regulators of pathways involved in apoptosis, acting to either inhibit or promote cell death (Reed, 1998). Bax can be regulated by p53 and show increased expression in certain tissues after apoptotic stimuli (Pena-Blanco and Garcia-Saez, 2017). In contrast with Bax, Bcl-2 is an anti-apoptotic protein. It was demonstrated that knockdown of Jab1 can mediate regulation of Bax and Bcl-2 (Sang et al., 2015). Upregulated of Bax and downregulated of Bcl-2 were observed when Jab1 was knocked down in AGS and MKN45 cells (Sang et al., 2015), which may explain the decreased cell proliferation and the increased apoptosis. Whether the regulation of Bax and Bcl-2 is controlled by the ubiquitin ligase activity or other ways remains to be determined.

## Biological significances of Jab1/COPS5

### Jab1/COPS5 in cell proliferation and cell cycle progression

Jab1/COPS5 is a major driving force for cell cycle by promoting degradation of several cyclins and CKIs (Shackleford and Claret, 2010; Yoshida et al., 2010). P27 is a universal CKI that directly inhibits the enzymatic activity of cyclin-Cdk complexes, resulting in cell-cycle arrest at G1 phase. Indeed, accumulating evidence has shown the inverse association between Jab1/COPS5 and p27 expression in various human malignancies, including HCC (Berg et al., 2007), NPC (Pan et al., 2012), oral squamous cell carcinomas (Gao et al., 2012) and papillary thyroid carcinoma (Ahn et al., 2009). Jab1/COPS5 negatively regulates p27 expression by exporting p27 from the nucleus to the cytoplasm, mediating p27 degradation via the proteasome pathway and promoting cell-cycle progression (Tomoda et al., 1999). Knockdown of Jab1/COPS5 results in the accumulation of p27 in cell nucleus, induces cell-cycle arrest and inhibits cell proliferation (Pan et al., 2012). It was also reported in a small number of cases that high Jab1/COPS5 expression had no correlation with low p27 expression (Schutz et al., 2012). One possible reason for these discrepancies could be tissue or cell type-specific differences. In addition, other factors, such as the ubiquitin ligase, Skp2, also contributes to the regulation of p27 (Carrano et al., 1999). In fact, regulation of p27 levels is complex, and more detailed functional studies may be warranted to explore this issue further. Expression of many other cell-cycle regulators, such as cyclin D, p16, p21, c-myc, and Bcl-XL (a member of the Bcl-2 family proteins), may also be regulated by Jab1/COPS5 (Shackleford and Claret, 2010). For example, Doronkin et al. demonstrated that depletion of Jab1/COPS5 induced cyclin E stability whereas Jab1/COPS5 overexpression rapidly degraded cyclin E (Doronkin et al., 2003). It is undoubted that Jab1/COPS5 plays critical role in the regulating cell cycle.

### Jab1/COPS5 in apoptosis

Evidence has shown that embryos of Jab1/COPS5-null mice have an elevated p53 levels, indicating this protein is a crucial regulator of p53 stability (Tomoda et al., 2004). In addition, targeted disruption of Jab1/COPS5 in mice leads to early embryonic lethality owing to accelerated apoptosis (Tian et al., 2010). Moreover, Jab1/COPS5 depletion results in more cisplatin-, ionizing radiation (IR)-, or ultraviolet (UV)-induced apoptosis, which was correlated with accumulated p53 in NPC (Pan et al., 2013b). Likewise, Sang et al. have reported that inhibition of Jab1/COPS5 promoted the apoptosis through p53-related apoptotic pathways in gastric cancer cells (Sang et al., 2015).

Jab1/COPS5 is also involved in many other apoptotic related pathways. For example, depletion of Jab1/COPS5 resulted in aberrant expression of the apoptosis-triggering protein Fas ligand in pro-B cells (Sitte et al., 2012). By specifically interacting with Jab1/COPS5, fibronectin type III and ankyrin repeat domains 1 (Fank1) inhibits cell apoptosis by activating the AP-1 induced anti-apoptotic pathway (Wang et al., 2011). In contrast, coexpression of BclGs (Bcl-Gonad short form) and Jab1/COPS5 synergistically promotes apoptosis since Jab1/COPS5 could compete with Bcl-XL/Bcl-2 to interact with BclGs (Liu et al., 2008). Jab1/COPS5 promotes E2F1-dependent induction of apoptosis as an E2F1-specific binding protein. Cells depleted of Jab1/COPS5 are deficient in both E2F1-induced apoptosis and p53 accumulation (Lu et al., 2011).

### Jab1/COPS5 in DNA damage response

Instability of genome caused by DNA damage can issue in cell cycle arrest and apoptosis. Loss of genomic integrity due to inactivation of DDR genes may induce accumulating gene mutation, which may greatly contributes to cancer development. Increasing evidence suggests that Jab1/COPS5 affects both the activity and stability of proteins involved in DDR (Pan et al., 2014). Jab1/COPS5 is crucial for tumor survival and enhances the resistance of tumor cells to chemotherapy and radiotherapy (Pan et al., 2013b). Importantly, Jab1/COPS5 promotes several DDR-related proteins' nuclear export and degradation. CSN can regulate the ubiquitinligase activity of the damaged DNA binding protein 1 (DDB1) or DDB2 and cockayne syndrome group A complexes (CSA) in response to damaging reagents. Loss of Jab1/COPS5 impaired the repair vitality of DDB2 (Groisman et al., 2003). Besides, Jab1/COPS5 also regulates Cdc10-dependent transcript 1 (CDT1) which is degraded after DNA damage. Jab1/COPS5 deficiency may block the degradation and lead to accumulation of CDT1 (Higa et al., 2003).

The Rad9-Rad1-Hus1 (9-1-1) complex is a DNA-damage sensor and plays an important role in initiating cellular responses to DNA damage. The 9-1-1 complex promotes ATR-mediated phosphorylation and activation of the Chk1, a protein kinase that regulates S-phase progression, G2/M arrest, and replication fork stabilization (Parrilla-Castellar et al., 2004). Jab1/COPS5 is involved in mediating the translocation and regulating the stability of the 9-1-1 complex in cells, which provides novel information about Jab1/COPS5's role in cell cycle checkpoint and DDR.

Rad51, a key DNA-repairing protein, plays an important role in DDR by facilitating production of nucleoprotein filaments and mediating strand conversion between DNA duplexes (Shinohara et al., 1992). Jab1/COPS5 has been shown to interact with Rad51 in homologous recombination (HR) repair pathway (Shinohara et al., 1992). Inhibition of Jab1/COPS5 not only decreases Rad51 level but also impaired its activity, resulting in an increase in cell apoptosis after DNA stimulus. Further studies demonstrated that Jab1/COPS5 regulates Rad51 through p53 pathway (Pan et al., 2013b).

### Jab1/COPS5 in reactive oxygen species (ROS) regulation

Cancer cells generate ROS as a result of stimulation of oncogenes, mitochondrial dysfunction, abnormal metabolism, and the microenvironment. Oncogenic mutations promote aberrant metabolism and protein translation, resulting in increased ROS production. Recent studies have begun to shed light on the ROS involved in modulating biological cell functions, cell signaling, and homeostasis in tumor cells (Shinohara et al., 2014). For example, acute myelocytic leukemia (AML) patients with FMS-like tyrosine kinase 3 (FLT3) mutations have a very high relapse rate (Gaballa et al., 2017), and FLT3 induced elevated levels of ROS (Sallmyr et al., 2008). Increased production of ROS or an inefficient antioxidant system leads to oxidative stress, which influences tumorgenesis. We recently investigated gene expression under oxidative stress in leukemia cell lines and blood samples obtained from AML patients at primary diagnosis and compared those with AML cells from the same patients obtained at relapse. High expression levels of Jab1 and thioredoxin (Trx) were associated with disease progression and poor prognosis in relapsed AML-M5. Mechanistically, Jab1 interacts with and positively regulates Trx expression. Increased ROS level stimulates aberrant gene expression and promotes the proliferation of leukemic blasts. We identified Jab1 as a new target of the ROS pathway that is implicated to have a role in the relapse and progression of malignant AML-M5. Specifically, we demonstrated that Jab1 interacts with and positively regulates Trx expression under oxidative stress (Zhou et al., 2017).

### Jab1/COPS5 in hypoxia

When cells are exposed to hypoxia, they either die or adapt to the hypoxic conditions. Both processes involve upregulation of the transcription factor hypoxia inducible factor-1 (HIF-1). One interesting function of Jab1 is its ability to promote oncogenesis by stabilizing HIF-1α. Importantly, Jab1 has been shown to interact directly with the CODD (C-terminal oxygen-dependent degradation domain) of HIF-1α as well as the pVHL E3 ligase (Bae et al., 2002; Bemis et al., 2004). Overexpression of Jab1 is associated with cancer progression and could reflect the deregulation of aerobic HIF stability, which is thought to be an important transition in tumor progression.

As another possible explanation for HIF-1 stability regulated by Jab1, the association between CSN and HIF-1 could be suggested. Jab1 may serve as a docking site for CSN complex-mediated phosphorylation and subsequently result in stabilizing HIF-1 protein. Additionally, CSN complex phosphorylates p53 at Thr-155 residue, and the phosphorylated p53 is subsequently degraded through ubiquitin-proteasome pathway rapidly (Bech-Otschir et al., 2001), suggesting that p53 degradation by CSN may lead to up-regulation of HIF-1 protein.

While Jab1 promotes HIF-1α stability and transactivation, p53 degrades HIF-1a and decreases its transactivation (Ravi et al., 2000; Hansson et al., 2002). Correspondingly, HIF-1a stabilizes p53, promoting apoptosis in this way (An et al., 1998; Chen et al., 2003). The binding of either Jab1 or p53 may also control the dephosphorylation of HIF-1α during an extended period of hypoxia. Thus we concluded that the interaction of Jab1 with HIF-1a promotes adaptation to hypoxia, in contrast to the interaction of p53 and HIF-1a at the same domain, which promotes apoptosis.

### Jab1/COPS5 in senescence

Cellular senescence is implemented in response to severe cellular insults such as oncogenic activation or chemotherapy and is a failsafe program that protects organismic integrity by excluding potentially harmful cells from further expansion (Narita and Narita, 2017). Senescence has been shown to cancel the pro-tumorigenic potential of Ras-/Raf-driven cancerous lesions (Braig et al., 2005; Michaloglou et al., 2005), and to contribute to the outcome of anticancer chemotherapy *in vivo* (Michaloglou et al., 2005).

It was found that a significant population of Jab1-depleted cells exhibited a senescence-associated heterochromatin foci (SAHF)-like structure regardless of the absence or presence of p53, indicating that Jab1-depletion initiated a senescence program independently on p53 (Yoshida et al., 2010). The function in the absence of p53 occurred with no significant change in the level of Skp2, which was implicated in the regulation of senescence (Lin et al., 2010). Further studies revealed that depletion of Jab1 inhibited cell proliferation, and induced premature senescence characterized by upregulation of senescence-associated- β-galactosidase activity and increased expression of CDK inhibitors (Tsujimoto et al., 2012). Jab1 was found to interact with CDK2 *in vivo* and *in vitro* (Yoshida et al., 2013). Depletion of Jab1 enhanced the phosphorylation of CDK2 by Akt, resulting in cytoplasmic accumulation of CDK2 together with cyclin E. Additional knockdown of CDK2, which reduced the expression of cyclin E to normal level, did not restore cell proliferation, but significantly inhibited senescence in Jab1- deficient cells. Enforced expression of cytoplasmic cyclin E induced premature senescence in immortalized cell lines (Yoshida et al., 2013). These results demonstrate that Jab1 functions through CDK2 to control premature senescence in a novel way (Yoshida et al., 2013).

It will be promising to explore small molecules which inhibit interaction between Jab1 and CDK2 but don't affect the deneddylase activity of Jab1, which may induce senescence without affecting global proliferation and the cell cycle, resulting in the development of anti-cancer drugs with minimum side effects.

## Clinical implication

### Efficiency of Jab1/COPS5 in combination with immunotherapies

Tumor cells can evade immune surveillance through expression of the inhibitory programmed cell death-ligand 1 (PD-L1) on the cell surface. However, the mechanisms by which PD-L1 is regulated have not been fully elucidated. Recently, researchers have investigated antitumor immunity in mouse model, demonstrating that inflammation promoted tumor growth and enhanced the number of tumor-infiltrating lymphocytes and macrophages, but decreased the cytotoxic activity of T cells. Inflammatory cytokines released by macrophages induced upregulation of PD-L1 protein in breast cancer. Mechanistically, inflammation induced macrophages to secrete TNFα that activated tumor cell NF-κB signaling, allowing the p65 subunit to bind to the promoter and promote transcription of Jab1/COPS5. Jab1/COPS5 encodes a deubiquitinating enzyme that was able to deubiquitinate and to stabilize PD-L1, leading to increased PD-L1 expression in response to inflammatory TNFα signaling. Further, the Jab1/COPS5 inhibitor curcumin blocked TNFα-induced PD-L1 stabilization in various cancer cells. Jab1/COPS5 expression was correlated with expression of PD-L1 in breast cancer. Moreover, increased Jab1/COPS5 expression was correlated with worse survival in breast cancer patients. Curcumin enhanced the therapeutic efficacy of anti-CTLA4 in mice, leading to decreased tumor growth and enhanced survival. Furthermore, curcumin plus anti-CTLA4 was effective in inhibiting tumor growth (Lim et al., 2016). These results suggest that Jab1/COPS5 stabilizes PD-L1 to promote tumor immune evasion and that suppression of Jab1/COPS5 may be effective in combination with immunotherapy.

### Therapeutic role of Jab1 in cancer

Increasing studies have suggested that Jab1/COPS5 is a novel therapeutic target given its prominent functions in different stages of tumorigenesis. Since Jab1/COPS5 overexpression is usually correlated with poor prognosis, development of Jab1/COPS5-specific inhibitors is likely to have a significant effect on cancer therapy (Table 1).

**Table 1:** Overview of the included studies and the results of Jab1/Cops5 inhibitors.

**Name**	**Indications**	**Effect**	**Reference**
Thiolutin	Neurospora and HeLa cells	CSN5 activity	Lauinger et al., 2017
Azelaic Acid	Acute Myeloid Leukemia	Protein level	Pan et al., 2017a
Azaindoles	HCT116 cells	Protease activity	Altmann et al., 2017
Curcumin	Breast cancer	Protein level	Lim et al., 2016
CSN5i-3	Various cancer cells	CSN5 activity	Schlierf et al., 2016
Curcumin analog T83	Nasopharyngeal carcinoma	Protein level	Pan et al., 2013a

Curcumin is a yellow plant pigment that has been shown to directly inhibit the activity of CSN associated kinases, making tumor cells arrest at the mitotic phase and prone to apoptosis through the inactivation of CSN (Fullbeck et al., 2005). It has been reported that polyethylene glycosylated curcumin (PEGylated curcumin), a water-soluble compound, inhibited pancreatic cancer cell growth by inhibiting Jab1/COPS5. Importantly, this compound induced sensitivity of pancreatic cancer cells to gemcitabine (Li et al., 2009). A novel curcumin analog T83 also significantly induced G2/M arrest and apoptosis in NPC cells in a dose- and time-dependent manner. In addition, T83 was effective in inhibiting Jab1/COPS5 expression and sensitizing NPC cells to radiotherapy (Pan et al., 2013a).

Another potential target drug is troglitazone which suppress Jab1/COPS5 promoter activity by inhibiting Sp1- and Tcf4-mediated Jab1 transcription (Hsu et al., 2008b). Both *in vitro* and *in vivo* studies have shown that troglitazone attenuates tumor growth and up-regulates p27 level effectively in a time- and dose-dependent manner (Motomura et al., 2000; Yu et al., 2006). Animal studies confirmed that troglitazone inhibited hepatocellular carcinima cell growth and decreased Jab1/COPS5 expression in tumor tissues (Hsu et al., 2008b).

As we know, the isopeptidase activity of Jab1/COPS5 resides in the JAMM motif, a zinc metalloproteinase-like domain which can be blocked by Zn^2+^ chelators (Echalier et al., 2013). Noteworthy, the JAMM motif of Jab1/COPS5 is an attractive therapeutic target because there is a plenty of experience in pharmacologic inhibition of metalloproteinases using small-molecule drugs, including popular antihypertensive drugs such as captopril and benazepril (Nalepa and Wade Harper, 2003). For example, Axel Diernfellner group recently demonstrated that Thiolutin, a disulfide-containing antibiotic and anti-angiogenic compound produced by *Streptomyces*, inhibits the JAMM metalloproteases of Jab1/COPS5 (Lauinger et al., 2017).

Others have provided insights into peptidomimetic antagonists for Jab1/COPS5 specific inhibitors since the C-terminal tail of one molecule docks in the active site of another one (Echalier et al., 2013). Anita Schlierf and colleague conducted a high-throughput screening campaign using a biochemical assay with recombinant CSN and a fluorescence-labeled and NEDD8-modified CRL substrate. They discovered a potent, selective and orally available Jab1/COPS5 inhibitor CSN5i-3. The compound traps CRLs in the neddylated state, which leads to inactivation of a subset of CRLs by inducing degradation of their substrate recognition module. CSN5i-3 was shown to differentially affect the viability of tumor cell lines and suppress the growth of a human xenograft in mice (Schlierf et al., 2016). These results provide insights into how CSN regulates CRLs and suggest that Jab1/COPS5 inhibition has potential for anti-tumor therapy.

**Figure 5:**
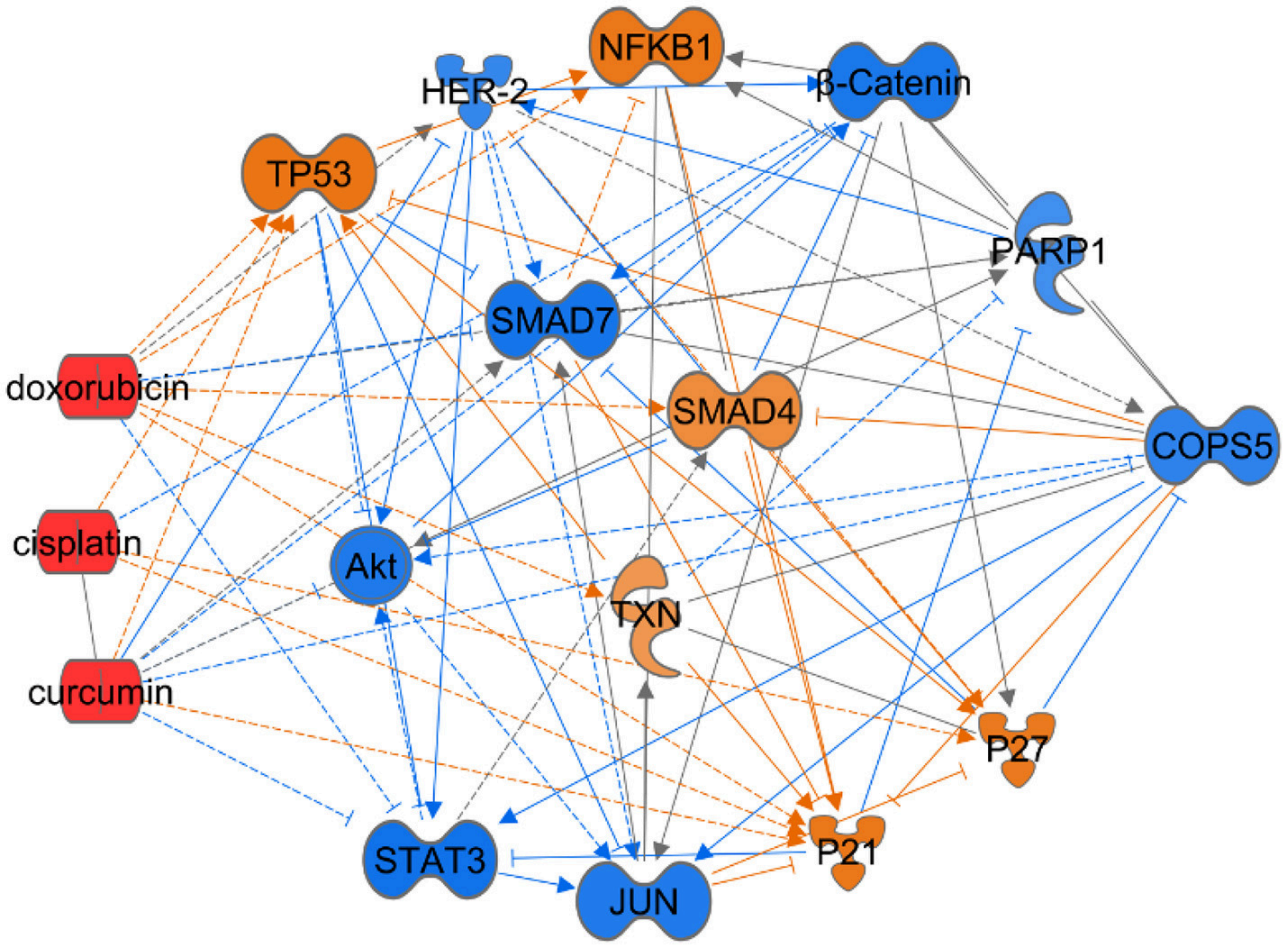
Ingenuity Pathway Analysis to predict the effect of chemical drugs on level of Jab1/COPS5. In this case we showed the predicted expression level of genes in the regulation network in response to increased level of doxorubicin, cisplatin, or curcumin based on the IPA database. The color of each gene represents the corresponding expression level. Light-red means activation and light-blue means inhibition, while red means increased measurement and green means decreased measurement.

Increasing evidence has supported Jab1/COPS5 as a therapeutic target and a network may exist between chemotherapy and Jab1/COPS5 (Figure 5). However, efforts are still needed to develop effective Jab1/COPS5 specific inhibitors suitable for clinical therapy. The degree, duration, and tissue specificity required for Jab1/COPS5 blockade to inhibit cancer growth need to be deeply explored.

## Jab1/COPS5 as biomarker for diagnostic and prognosis

Jab1/COPS5 has been observed to overexpressed in many cancers including colon, ovarian, lung and breast cancer, and so on (Pan et al., 2014). Jab1/COPS5 expression is associated with tumor progression and outcomes in many cancer patients. Recently, we retrospectively collected and analyzed data of 88 lung cancer and 76 breast cancer patients, and found that lung cancer patients with higher Jab1 level was less responsive to chemotherapy. Recurrent breast cancer patients had much higher Jab1 level (Hou et al., 2017). Both breast cancer patients and lung cancer patients with higher Jab1 level had significantly shorter disease-free survival and overall survival. In a multivariate survival analysis, Jab1 was correlated with disease-free survival and overall survival in breast cancer (Hou et al., 2017). The Jab1 level was found to be a possible biomarker for clinical response to chemotherapy in lung cancer patients and for postoperative relapse in breast cancer patients who received adjuvant chemotherapy (Hou et al., 2017), indicating that Jab1 predicts treatment response in lung cancer and relapse in breast cancer patients.

## Conclusion and overall perspectives

In this review, we briefly illustrate the structure and functions of Jab1/COPS5. With its multiple prominent functions, Jab1/COPS5 affects various targets and signaling pathways, which are normally carcinogenic. Jab1/COPS5 has been proved to be associated with therapeutic response and adverse outcome in cancer patients. Although the exact underlying mechanism is inexplicit, it provides a new research direction of anticancer treatment. As a target of anticancer therapy, more further studies of Jab1/COPS5 will promote the development of novel therapeutic strategies against cancer. Considering the pivotal cellular role of Jab1/COPS5, it is reasonable to assume that Jab1/COPS5 is a promising diagnostic and prognostic biomarker for cancer in the future.

Furthermore, we summarized several compounds that could target Jab1 and may act as anti-cancer drugs. Due to non-toxic features, inhibition of Jab1 by natural agents would be a novel and safe approach for cancer therapy. However, further pre-clinical research is urgent to find the right combinations with chemotherapy or radiotherapy toward better treatment of human cancers. We hope this article could promote further studies for the development of specific inhibitors for Jab1 in cancer treatment by either single agent or by a combinational approach. Collectively, targeted inhibition of Jab1 would become a novel strategy for the prevention of tumor progression and successful treatment of human cancers in the future.

## Author contributions

YP: conceived the study; GL and YP: analyzed data and wrote original draft; FZ and FXC: reviewed and edited the manuscript.

### Conflict of interest statement

The authors declare that the research was conducted in the absence of any commercial or financial relationships that could be construed as a potential conflict of interest.
